# Sustained, Controlled and Stimuli-Responsive Drug Release Systems Based on Nanoporous Anodic Alumina with Layer-by-Layer Polyelectrolyte

**DOI:** 10.1186/s11671-016-1585-4

**Published:** 2016-08-23

**Authors:** Maria Porta-i-Batalla, Chris Eckstein, Elisabet Xifré-Pérez, Pilar Formentín, J. Ferré-Borrull, Lluis F. Marsal

**Affiliations:** Department of Electronic, Electric and Automatics Engineering, Universitat Rovira i Virgili, Avda. Països Catalans 26, 43007 Tarragona, Spain

**Keywords:** Drug delivery, Electrochemical anodization, Nanoporous alumina, Stimuli-responsive release, Doxorubicin

## Abstract

Controlled drug delivery systems are an encouraging solution to some drug disadvantages such as reduced solubility, deprived biodistribution, tissue damage, fast breakdown of the drug, cytotoxicity, or side effects. Self-ordered nanoporous anodic alumina is an auspicious material for drug delivery due to its biocompatibility, stability, and controllable pore geometry. Its use in drug delivery applications has been explored in several fields, including therapeutic devices for bone and dental tissue engineering, coronary stent implants, and carriers for transplanted cells. In this work, we have created and analyzed a stimuli-responsive drug delivery system based on layer-by-layer pH-responsive polyelectrolyte and nanoporous anodic alumina. The results demonstrate that it is possible to control the drug release using a polyelectrolyte multilayer coating that will act as a gate.

**Electronic supplementary material:**

The online version of this article (doi:10.1186/s11671-016-1585-4) contains supplementary material, which is available to authorized users.

## Background

Nearly 90 % of the existing drugs are hydrophobic which means they cannot be dissolved in the blood. This reduces their pharmacological efficiency. On the other hand, some bioactive agents such as proteins, nucleic acids, or enzymes administered though oral or intravenous routes can be easily degraded by metabolism or by enzymatic conditions and are unable to reach the desired sites [1–3]. Increasing the knowledge of materials at the nanoscale may accelerate the improvement of drug delivery systems, especially in treating life-threatening conditions such as cancer and heart disease. Nanoporous and nanotube carriers with their unique features such as low-cost fabrication, controllable pore/nanotube structure, tailored surface chemistry, high surface area, high loading capability, chemical resistivity, and mechanical rigidity have affianced a special role in drug delivery technology. Drug release is a process in which a composite or a device releases a drug in a controlled way and is subjected to absorption, distribution, metabolism and excretion (ADME), finally becoming available for pharmacological action. To achieve and preserve therapeutically effective plasma concentrations, several doses are needed daily, which may cause significant fluctuations in plasma levels. Because of these fluctuations in drug plasma levels, the drug concentration could fall below the minimum effective concentration or exceed the minimum toxic concentration. Such changes result in undesirable side effects or lack of therapeutic profit to the patient.

Sustained-release and controlled-release drug delivery systems can reduce the undesired fluctuations of drug levels, consequently diminishing side effects while improving the therapeutic result of the drug. The terms sustained release and controlled release refer to two different kinds of drug delivery systems (DDS), although they are often used interchangeably. Sustained-release dosage forms are systems that elongate the duration of the action by reducing the release of the drug and its pharmacological action. Controlled-release drug systems are more sophisticated than just simply delaying the release rate and are designed to deliver the drug at specific release rates within a predetermined time period. Advantages of controlled release DDS comprise delivery of a drug to the required site, maintenance of drug levels within a desired range, reduced side effects, fewer administrations, and improved patient compliance. The evolution of delivery systems leads to stimuli-responsive DDS, whose behavior can be dependent on the environment where it is applied. In recent years, the pH-responsive controlled drug delivery systems have attracted considerable attention because of the acidic tumoral environment of most cancers and the acidic environs of wounds [4]. In this work, we propose a DDS that can be defined as a sustained, controlled and stimuli-responsive release system due to its capability to release the drug in a desired rate and responding to pH changing stimulus.

The DDS we propose is based on nanoporous anodic alumina (NAA). It was not until the 1990s that researchers discovered that highly ordered nanoporous structures can be achieved by properly tuning anodization conditions including electrolyte composition and concentration and temperature, as well as anodization voltage [5]. Some studies have been already performed in the drug delivery framework using porous materials [6–8]. Nanoporous anodic alumina is one of the most attractive materials for drug delivery applications since it has simple and low-cost fabrication and the pore size and depth can easily be controlled by regulating the anodizing voltage, time, and electrolyte composition. Other remarkable properties of this material are the chemical and thermal stability, hardness, high surface area, and highly ordered pore structure [9, 10]. Some applications of NAA are to reconstruct or regenerate living tissues and deal with infections and inflammation as consequence of chirurgical implantation or just for drug constant administration [11]. Drug depots in the human body with controlled and retained release are able to improve quality of life and assist long-term treatments. In addition, the development of those new and more efficient drug delivery systems solve conventional drug therapy problems related to limited drug solubility, lack of selectivity, and unfavorable pharmacokinetics.

The structure of NAA can be described at a close-packed hexagonal and perpendicular orientated array of columnar cells, each containing a central pore, of which the size and interval can be controlled by changing the anodization conditions. The drug release from porous materials is based on molecular diffusion from the pores, and it is mainly governed by the pore dimensions [12]. Therefore, adjustment of pore diameter and pore depth has been considered a common strategy to control drug release performance.

In this study, NAA platforms with a pore diameter of 130 nm and pore depth of 15 μm were used as a model porous material. In order to realize a controlled drug release, a pH stimuli-responsive polyelectrolyte layer-by-layer (LbL) assembly has been used to coat the porous matrixes. Doxorubicin (DOX), a potent antineoplasic agent against a wide range of human tumors, was chosen as a model drug to perform the trials. The polyelectrolyte multilayer on the surface prevents the early release of the drug and enables the use of the total enhanced surface in the NAA samples. The effect of pH in the drug release kinetics has been studied and discussed as well as the effect of the polyelectrolyte bilayer number.

## Methods

### Nanoporous Alumina Anodization

**Fig. 1:**
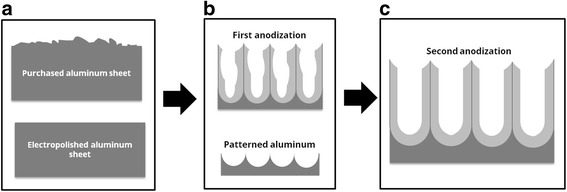
Schematic representation of the alumina pores formation during the anodization process. **a** The electopolishing procedure creates a plane surface. **b** The first anodization followed by the dissolution of the alumina wall creates an ordered pattern in the aluminum sheet. **c** The second anodization on the patterned aluminum creates a perfect ordered NAA

Ordered nanoporous anodic alumina was prepared by the two-step anodization method (Fig. 1) [13–15]. Aluminum plates were degreased in acetone and ethanol to eliminate organic impurities. They were then subsequently electropolished (Fig. 1a). Then they were anodized for the first time using a ramp to achieve the desired voltage. At that moment, the disordered porous alumina was removed by the wet chemical etching (Fig. 1b), leaving a highly periodic structure of nano-concavities [16–19]. At that time, a second anodization was performed obtaining an ordered and 15-μm deep alumina porous layer (Fig. 1c). This process is explained in detail in the supplementary information.

### Polyelectrolytes Assembly

**Fig. 2:**
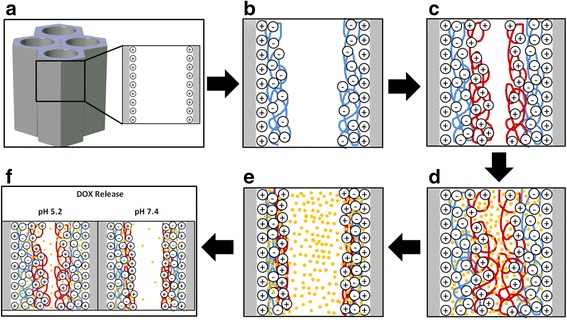
Schematic representation of the polyelectrolyte layer-by-layer deposition procedure. **a** NAA pores with positively charged walls after APTES treatment. **b** PSS deposition by immersing the APTES treated surface. **c** PAH deposition by immersing the PSS covered substrate. **d** DOX loading in the swollen PEM film at pH 2.0. **e** DOX confinement due to the PEM layer contraction at pH 8.0. **f** DOX releases at different pH media

In order to cover the nanopore walls with polyelectrolyte layers, nanoporous anodic alumina was first coated with 3-aminopropyl triethoxysilane (APTES). The positively charged APTES substrates would allow negatively charged polyelectrolytes to be attached to the pore walls (Fig. 2a). For LbL deposition, the NAA substrates were immersed consecutively into a negatively charged solution of poly(styrenesulfonate) (PSS, 1 mg/ml in 5 mM CaCl_2_ in deionized water) (Fig. 2b) and a positively charged solution of poly(allylamine hydrocloride) (PAH, 1 mg/ml in 5 mM CaCl_2_ in deionized water) (Fig. 2c), alternating rinsing with deionized water between each immersion. Dipping times in polyelectrolyte solutions were 30 min and the washing step in deionized water lasted for 10 min [20]. All the steps were repeated for two, five, and eight times for obtaining two, five, and eight bilayers, respectively.

### Drug Loading

Positively charged DOX hydrochloride was selected as a model drug. LbL NAA samples were immersed in 1 mg/ml DOX solution at pH 2 in the dark at room temperature overnight (Fig. 2d). Then the DOX solution was adjusted to pH 8 and the samples were stirred 2 h in the dark (Fig. 2e). Subsequently, samples were washed with deionized water at pH 8. At pH 2, the increased permeability of the polyelectrolytes film facilitates the incorporation of DOX inside the PSS/PAH multilayers. Then the adjustment of pH at 8 causes the contraction of the polyelectrolytes and the drug molecule becomes trapped inside the polyelectrolyte film. The following washing will remove any nontrapped DOX molecule.

### Drug Release

Samples under test were immersed in phosphate buffered saline (PBS) at pH 7.4 and sodium acetate buffer at pH 5.2 (Fig. 2f). Samples were immersed in 0.5 ml of the corresponding medium and this medium was renewed at every measurement. Release characteristics depending on the number of polyelectrolyte layers and on the pH of the release medium were examined. Release experiments consisted of monitoring the diffusion of DOX as a function of time after the encapsulation within the polyelectrolyte coating. For this reason, fluorescence of the buffers solutions was measured at regular time intervals. The photoluminescence measurements were taken on a fluorescence spectrophotometer with a Xe lamp used as the excitation light source at room temperature and an excitation wavelength of 480 nm. Drug release was monitored by drug photoluminescence over 7200 min (120 h) in two different pH buffer mediums: pH 5.2 and pH 7.4. Once we reached 2880 min (48 h), the pH 7.4 medium was changed for pH 5.4 medium. Intensities of the fluorescent peaks were converted to the corresponding concentrations using a calibration curve. Release rates are reported as μg/cm^2^ vs. time.

## Results and Discussion

**Fig. 3:**
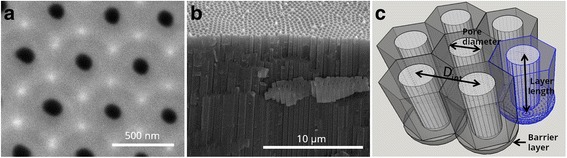
**a** Top view ESEM image of NAA. **b** Cross-sectional SEM image of imprint NAA. **c** Schematic representation of the alumina pores forming a close-packed hexagonal and perpendicular orientated array of columnar cells

Figure 3 shows environmental scanning electron microscopy (SEM) images of one of the fabricated NAA samples and a schematic drawing of the porous structure. The top surface view in Fig. 3a reveals a good ordering in a honeycomb structure of the pores in the short range, while the cross section in Fig. 3b demonstrates straight and parallel growth of the pores. Image analysis results in estimated average pore diameter (*d*_pore_) value of 150 nm, pore length (*L*_pore_) of 15 μm, and interpore distance (*D*_int_) of 480 nm. The schematic drawing in Fig. 3c illustrates the definition of these magnitudes.

**Fig. 4:**
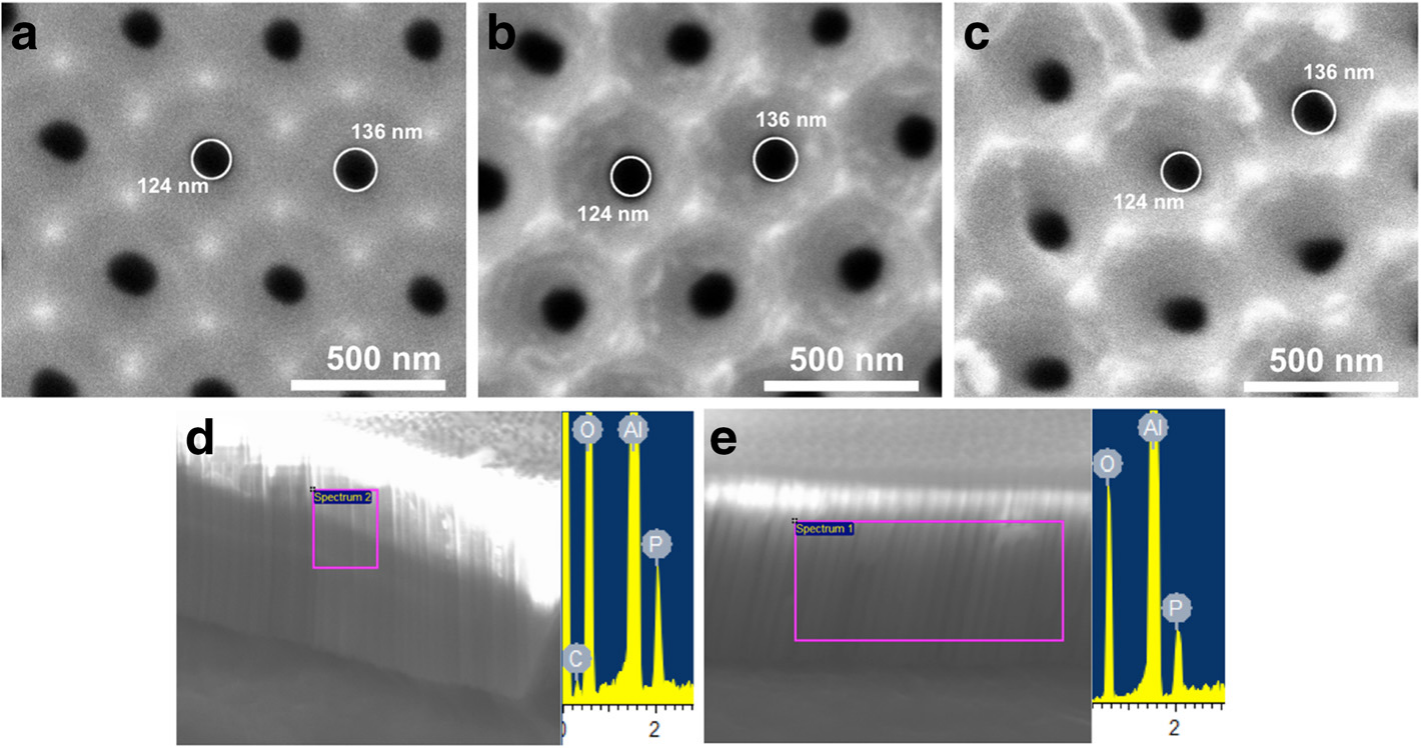
Environmental scanning electron microscope images of the top views **a** without polyelectrolyte coat, **b** with two polyelectrolyte bilayers, and **c** with eight polyelectrolyte bilayers. *Circles* about 124 and 136 are drawn in the images. The EDX measurements for cross section samples without polyelectrolyte coating (**d**) and with polyelectrolyte coating (**e**)

Figure 4 shows SEM pictures of the top surface of a NAA sample after different steps in the PSS/PAH deposition, in order to validate the successful deposition of the polyelectrolyte multilayer. Figure 4a corresponds to an as-produced sample, Fig. 4b to a sample after the deposition of two polyelectrolyte bilayers, while Fig. 4c corresponds to a sample after the deposition of eight polyelectrolyte bilayers. The pictures do not show a noticeable change in pore diameter. A statistical estimation of pore diameters using image processing techniques was carried out; the results are included in Additional file 1: Figure S2 A–C and Table S1. This statistical estimation results in an average pore radius of 130 nm for the three pictures in Fig. 4a–c with a standard deviation of 12 nm. To further illustrate the invariability in the pore diameter from the pictures, two circles are drawn on the figures corresponding to the maximum and minimum size obtained from this estimation. The only indication from the pictures that the surface is being properly modified is that the image contrast indeed increases with the number of bilayers. Hence, it can be assumed that there is a polyelectrolyte coat covering the sample surface. In order to confirm adequate infiltration and polyelectrolyte coating in the inner pore surfaces, we imaged a cross section of the nanopores before and after coating with polyelectrolytes and we obtained the energy-dispersive X-ray spectroscopy (EDX) spectra shown in Fig. 4d, e.

It can be assumed that no pore blockage occurred during the LbL self-assembly. The use of multivalent salt such as CaCl_2_ contributes to the formation of the polyelectrolyte layer inside the nanopore owing to a stronger polymer-chain contraction [21, 22]. The subsequent EDX analysis of those samples shows phosphoric and aluminum peaks due to the sample and electrolyte presence and also an oxygen peak because of the presence of this element in the alumina sample (Al_2_O_3_). However, a carbon peak only appeared on those samples with polyelectrolytes (Fig. 4d). That peak could not be found in the alumina samples without polyelectrolyte treatment (Fig. 4e). This observation confirms the successful deposition and insertion of both polyelectrolytes and DOX into the pores.

After the DOX loading, samples were exposed to different pH media to evaluate the pH responsiveness and influence of the number of polyelectrolyte bilayers. Once in contact with the aqueous medium, the polyelectrolyte multilayer swells to a certain extent, increasing its permeability and allowing the diffusion of the drug. The swelling mechanism of PAH/PSS films is generally associated to the difference in charge density of polyelectrolyte chains induced by a change in the pH medium. PAH is a weak polyelectrolyte whose amino groups become charged when the pH decreases, producing an increase in the osmotic pressure. Consequently, water molecules diffuse into the polyelectrolytes and the multilayer swells. This phenomenon, together with the electrostatic repulsion between DOX and PAH/PSS multilayer, enables the diffusion of the drug in the medium [23].

**Fig. 5:**
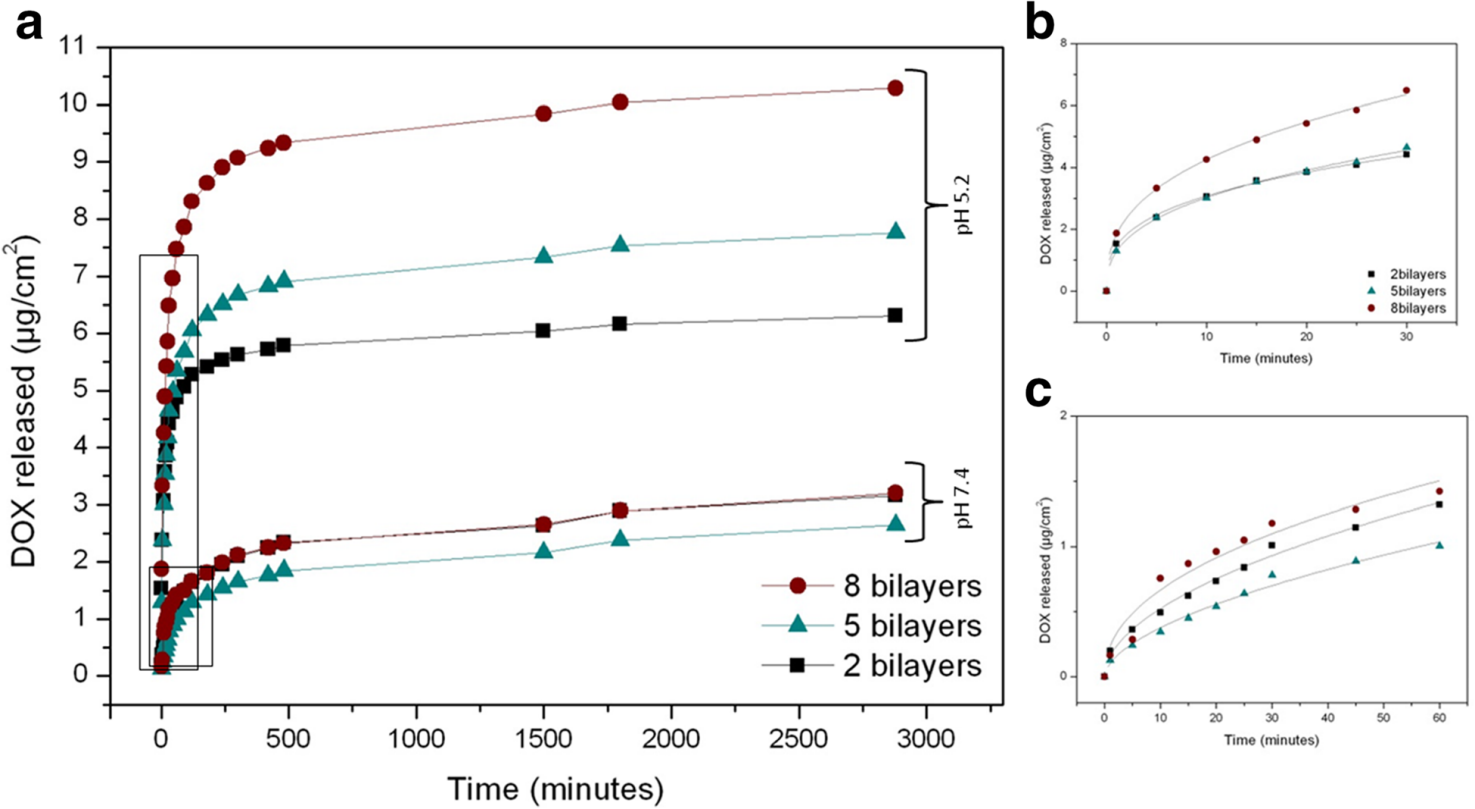
**a** Doxorubicin (DOX) release profile for 3000 min at pH 5.2 and 7.4 for different numbers of polyelectrolyte bilayers. **b** Nonlinear fitting for the burst release at pH 5.2. **c** Nonlinear fitting for the second burst release at pH 7.4

Figure 5a compares the release profile of DOX from samples with different number of layers at pH 5.2 and 7.4 over a period of 3000 min. As it can be seen, there are two groups of curves: one group at pH 5.2 and another group at pH 7.4. Each group contains three different curves: eight bilayer samples (circles), five bilayer samples (triangles), and two bilayer samples (squares). In general terms, it can be said that there is a massive burst release in all curves (framed in the graph) within the first minutes. Once this first stage has occurred, the release rate decreases causing a curve flattening.

Figure 5b, c shows a closer look for the burst releases at pH 5.2 and 7.4, respectively. The data show that (as expected) burst release at pH 5.2 is faster than burst release at pH 7.4. The results at pH 5.2 within the first 30 min (Fig. 5b) show that the samples with five and two bilayers release approximately the same amount of drug, while for the eight bilayer samples, the release is 1.4 times bigger. After stabilization, at pH 5.2, the amount of released drug is bigger for a bigger number of bilayers: samples with eight bilayers release 1.32 times more drug than five bilayer samples and 1.63 times more than two bilayer samples. Instead, at pH 7.4, the release dynamics is different: there is not a clear correspondence between the amount of released drug and the number of bilayers, both in the burst and in the sustained-release periods. These observed differences may occur due to the inhibition caused by the polyelectrolyte contraction.

**Fig. 6:**
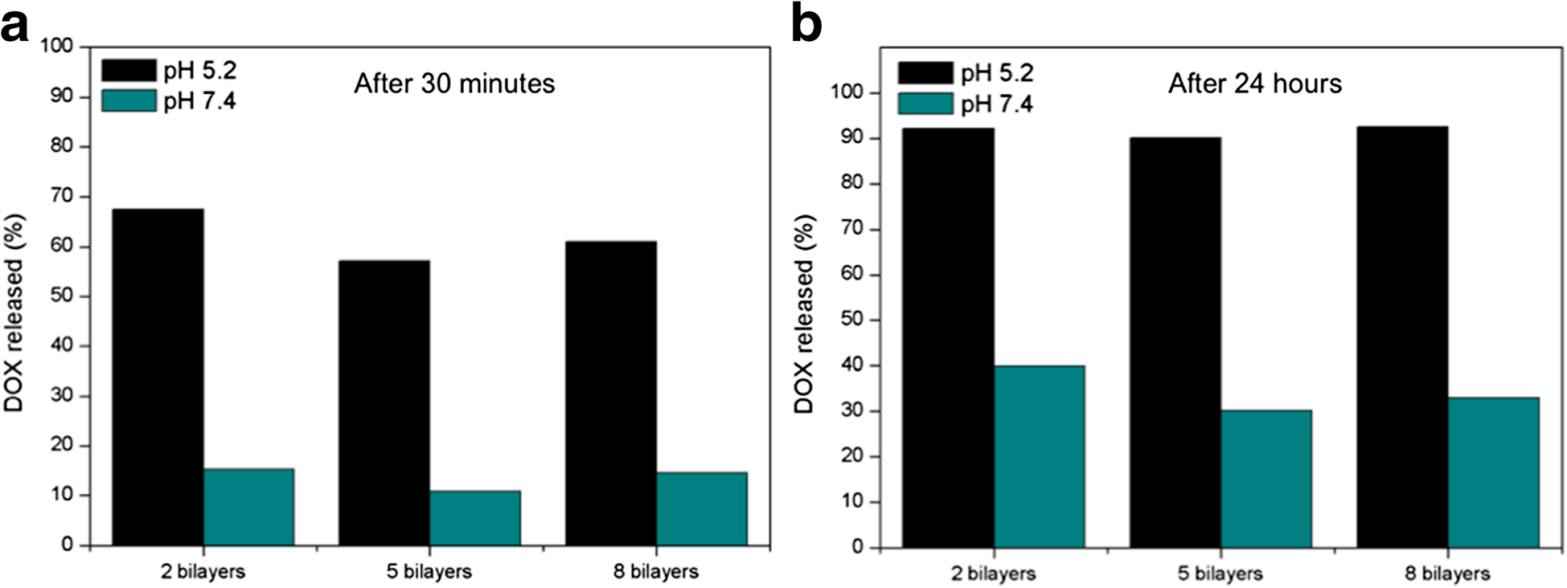
**a** Percentage of the DOX released within the first 30 min at different pH and bilayer number. **b** Percentage of the DOX released after 24 h for different pH and bilayer numbers

Considering relative values, taking into account that 100 % of the drug is the total amount of drug released at infinite time, the DOX released after 30 min for samples at pH 5.2 is between four and five times higher than that at pH 7.4 (Fig. 6a). The result is in accordance with the result in Fig. 5. In addition, the relative amount of released drug is not depending on bilayer number: 90 % of the drug has been released during the first 24 h at pH 5.2 while only 30–40 % of the drug is released within first 24 h at pH 7.4 (Fig. 6b). At that time, release rate is reduced gradually until it shows a stabilized profile. We can observe that at longer times the difference between relative released DOX at pH 7.4 and pH 5.2 is becoming lower. Since the liberation of the drug at pH 7.4 is slower, it is also more sustained during time. This is the reason why the total amount of drug release at pH 5.2 and 7.4 is becoming closer.

**Fig. 7:**
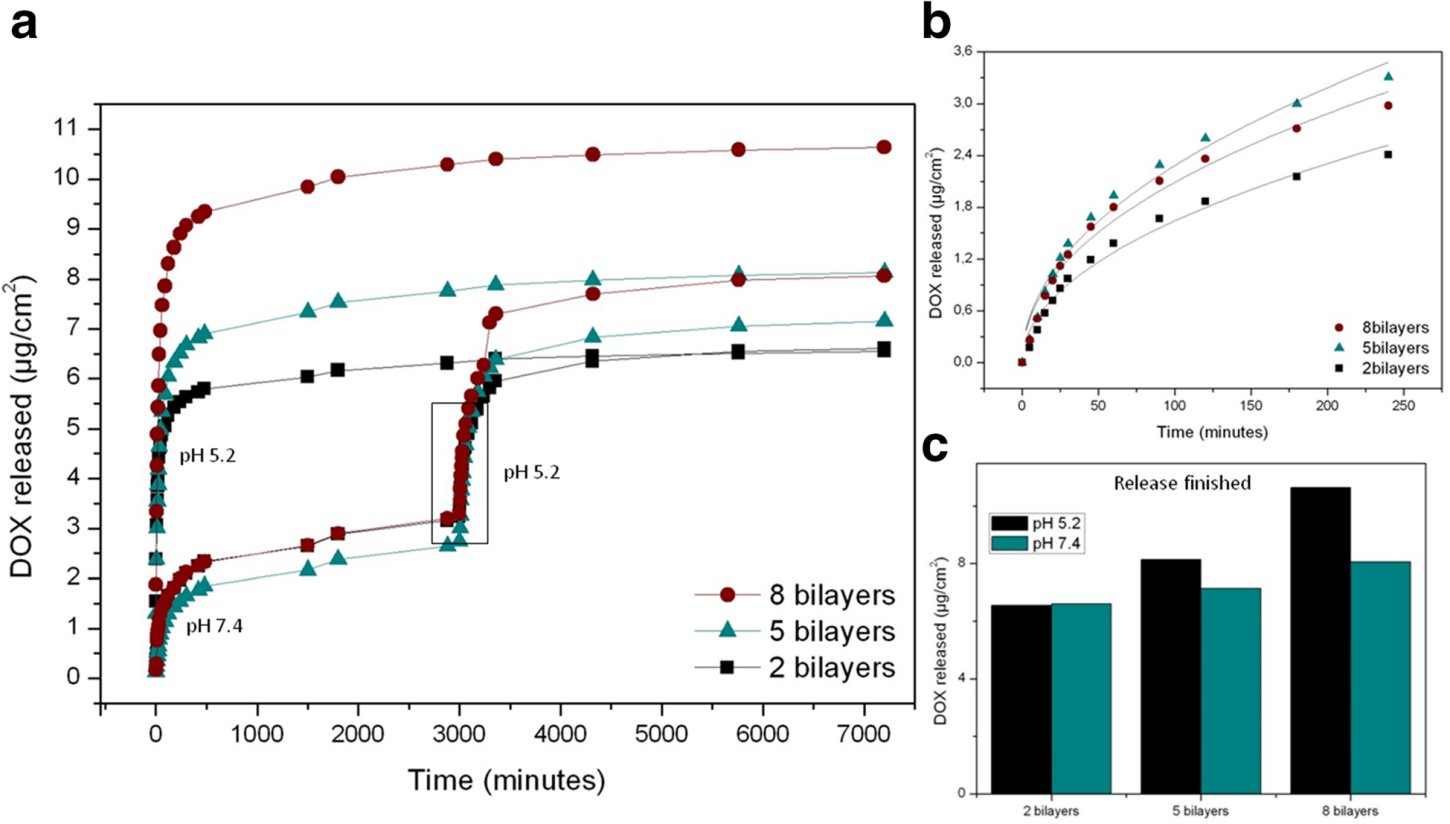
**a** Complete release profiles of DOX from NAA coated with different polyelectrolyte bilayer numbers at pH 5.2 and 7.4 with different burst releases framed. **b** Nonlinear fitting for the second burst release at pH 5.2. **c** Total DOX amount released for every different sample during the monitoring

In Fig. 7a, a general profile of the release is shown in order to prove the responsiveness of the DDS to pH variation. At minute 3000, samples immersed with the medium at pH 7.4 were immersed to a pH 5.2 medium. This change in pH triggers another burst release really similar to the first burst release in samples at pH 5.2, which demonstrates that the DDS responds to pH modification. The amount of drug released after the stabilization in the second burst release at pH 5.2 correlates with the number of bilayers. However, the absolute amount of DOX released in this second burst release is not reaching the same values of the first burst release at the same pH for all the samples. Specifically, for two bilayers, the drug released reaches the same value as with the previous release at pH 5.2. Instead, for five bilayers, the total amount only reaches up to 87.5 % of the drug released in the previous experiment at pH 5.2, while for eight bilayers, this percentage is even lower (72.7 %).These results can also be noticeably seen in Fig. 7c.

Figure 7b displays a detailed fitting for the second burst release at pH 5.2. And Fig. 7c shows a comparison between the total amounts of DOX at the finished release time for the different samples. In addition, total amount of encapsulated DOX was also studied concluding that there is a proportionally direct relation between the number of polyelectrolyte bilayers and the amount of DOX released (Fig. 7c). This relation can be observed in both pH mediums but becomes more obvious at pH 5.2 when DOX molecules can diffuse with less obstruction.

In order to perform a quantitative analysis of the results during the initial stage (burst release), we performed a fitting study of the curves by a variation of the Higuchi and Ritger-Peppas models. The Higuchi model is an empirical model commonly used to describe the release kinetics of drugs from insoluble porous materials [24, 25] It is well established and commonly used for modeling drug release from matrix systems [25–27]. The model is based on a square root of time-dependent process of Fickian diffusion [28, 29]. Fick’s law of diffusion provides the fundamentals for the description of solute transport from matrices [30]. The Ritger-Peppas model (also known as Korsmeyer-Peppas) is used to fit drug release from polymeric thin films, cylinders, and spheres [31]. The used equation is:

$$ {M}_t={M}_{t_0}{\left(\frac{t}{t_0}\right)}^n, $$

where *M*_*t*_ is the proportion of DOX released at a given time *t*, ${M}_{t_0}$ is the amount of released drug at the reference time *t*_0_ (1 min), *t* is time in minutes, and *n* is a fitting parameter related to the release rate. The adjustment procedure using a least squares method minimizes the differences between the experimental and theoretical values [32–34]. Best fit values for those different parameters are reported in Table 1.

**Table 1:** Nonlinear fitting parameters for the different burst releases using the equation ${M}_t={M}_{t_0}{\left(\frac{t}{t_0}\right)}^n$

		${M}_{t_0}$	*n*	Release rate
First burst release, pH 5.2	8 bilayers	1.84 ± 0.05	0.36 ± 0.01	0.67
	5 bilayers	1.29 ± 0.04	0.37 ± 0.01	0.48
	2 bilayers	1.47 ± 0.05	0.32 ± 0.01	0.47
Burst release, pH 7.4	8 bilayers	0.23 ± 0.04	0.45 ± 0.05	0.11
	5 bilayers	0.10 ± 0.01	0.57 ± 0.03	0.06
	2 bilayers	0.16 ± 0.01	0.52 ± 0.02	0.08
Second burst release, pH 5.2	8 bilayers	0.24 ± 0.03	0.47 ± 0.03	0.11
	5 bilayers	0.25 ± 0.03	0.48 ± 0.03	0.12
	2 bilayers	0.17 ± 0.02	0.49 ± 0.03	0.08

The data in Table 1 is showing ${M}_{t_0}$, *n*, and release rate which has been obtained as the first derivative of the equation at time *t*_0_$\left({M}_{t_0}\times n\right)$. The values of ${M}_{t_0}$ for the first release at pH 5.2 are one order of magnitude higher than for the first release at pH 7.4, in good agreement with the behavior observed in Fig. 5. Furthermore, for pH 5.2, there is a clear difference between ${M}_{t_0}$ for eight bilayers on one hand, and ${M}_{t_0}$ for five and two bilayers on the other. This result suggests that the main contribution to the drug release at pH 5.2 is coming from the outer layers. Instead, for pH 7.4, the difference between the ${M}_{t_0}$ is much smaller, which leads to the conclusion that only the drug in the outermost layer is contributing to the release. These results are in good agreement with the influence of pH on the amount of released drug observed in Fig. 5. In what respects the value of *n*, it can be seen that the values for each pH are similar for the different number of bilayers. This indicates that the release dynamics is influenced by pH but not by the number of polyelectrolyte bilayers.

It is also interesting to note that for the second release at pH 5.2, the ${M}_{t_0}$ and the release rate are sensibly smaller than for the first release at pH 5.2. With this, it can be concluded that, although the DDS is sensitive to pH variation, the first release at pH 7.4 modifies the dynamics of further release events triggered by such pH variation. We attribute this fact to the availability of DOX within the polyelectrolytes. As part of the drug, mainly from the outermost layer, has been already released at pH 7.4, the remaining drug from deeper layers finds it more difficult to diffuse into the medium.

## Conclusions

Tubular NAA membranes coated with polyelectrolytes are presented as a stimuli-responsive pH-dependent drug delivery system (DDS). The membranes were fabricated using a two-step anodization process that resulted in a highly uniform pore size distribution. These membranes are coated with a pH-responsive polyelectrolyte and effectively loaded with DOX to evaluate the influence of pH and of the number of polyelectrolyte bilayers on the release dynamics. Higher total amounts for released DOX were found in samples immersed in acidic medium, confirming the pH responsiveness of the DDS. The amount of released DOX in acidic medium is in correlation with the number of polyelectrolyte bilayers, although the increase in released drug does not scale linearly with the number of polyelectrolyte bilayers. This suggests that only the outer bilayers in the polyelectrolyte structure contribute to the release at this pH. On the other hand, when release is performed at pH 7.4, the amount of released drug does not depend on the number of polyelectrolyte layers, which leads to the conclusion that only the drug nearest to the medium is released. The quantitative analysis of the release curves also revealed that the release dynamics (related with the exponent n in the Ritger-Peppas model) depends strongly on the pH, but the number of polyelectrolyte layers does not influence it. If an abrupt change in pH is applied to the DDS, from neutral to acidic medium, a second burst release is triggered. This second burst release shows a dynamics different than the first release at pH 5.2. This can be attributed to the limited availability of drug in the outermost layers, after the first release at pH 7.4. To conclude, results show that nanoporous anodic alumina coated with layer-by-layer pH-responsive polyelectrolyte has potential applications in local drug delivery.

## Additional file

Additional file 1: Supplementary information. (DOCX 481 kb)
