# Supplementary material for: Sustained, Controlled and Stimuli-Responsive Drug Release Systems Based on Nanoporous Anodic Alumina with Layer-by-Layer Polyelectrolyte
Source: Nanoscale Res Lett. 2016 Aug 23;11:372. doi: 10.1186/s11671-016-1585-4 (PMC4993726; doi:10.1186/s11671-016-1585-4)
Supplement: Additional file 1: — Supplementary information. (DOCX 481 kb) [file 11671_2016_1585_MOESM1_ESM.docx]

Supplementary Information

**SUSTAINED, CONTROLED AND STIMULLI-RESPONSIVE DRUG RELEASE SYSTEMS BASED ON NANOPOROUS ANODIC ALUMINA WITH LAYER-BY-LAYER POLYELECTROLYTE**

**
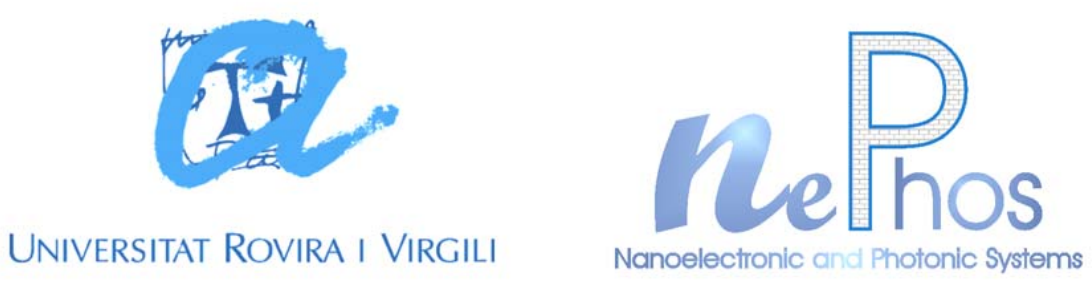
**

mAria porta-i-batalla, chris eckstein, Elisabet XIFRÉ-PéREZ, Pilar formentín, josep ferré-borrull, lluis f. marsaL

Nano-electronic and Photonic Systems (NePhoS)

Departament d’Enginyeria Electrònica, Elèctrica i Automàtica

Universitat Rovira i Virgili.

Avda. Països Catalans 26, 43007 Tarragona, Spain.

**Methods**

**Nanoporous alumina anodization**

Ordered nanoporous anodic alumina was prepared by the two-step anodization method (Figure 1). Aluminum plates were degreased in acetone and ethanol to eliminate organic impurities. They were then subsequently electropolished (Figure 1A) in a mixed solvent of perchloric acid and ethanol (1:3) at a constant applied voltage of 20 V for 6 min. To suppress breakdown effects and to enable uniform oxide film growth at high voltage (195 V in phosphoric acid) a protective layer at lower voltage (175 V in phosphoric acid) for 180 min was obteined. In order to have a more precise control of the anodization process in this work, the voltage and current of the anodization process were monitored and recorded by DSM (SM 300-5) SourceMeter, controlled by a home-built computer program based on LabView. After this pre-anodization at 175 V, a ramp of 0,05 V/s was used to reach the hard anodization voltage (195 V) during 24 hours. The voltage was increased in a linear manner, so that the current will not rise too fast in order to avoid overheating of the electrolyte and the subsequent oxide breakdown. Once the ramping process is done, the program mantains the target voltage while it is observed that the anodization current falls naturally down to a stable level, which is an indication of inception of stable anodization. Then, after this first step, the porous alumina grown on the aluminum surface was removed by a wet chemical etching in a mixture of phosphoric acid (0,4 M) and chromic acid (0,2 M) (1:1 volume ratio) at 70ºC, leaving a highly periodic structure of nano-concavities on the surface of the aluminum substrate (Figure 1B), which forms the initiation sites for the formation of pores in the second anodization step. The second anodization step was performed under the same experimental conditions (194 V) as in the first step in order to obtain ordered nanoporous alumina (Figure 1C). The second step anodization was applied until pores with 15 µm depth were obatined.. The morphologies of the NAA substrates were characterized by Environmental Scanning Electron Microscopy (ESEM FEI Quanta 600, Hillsboro, OR, USA).

**Figure 1: Calibration curve for different Doxorubicine concentrations and their Photoluminescence values.**

**
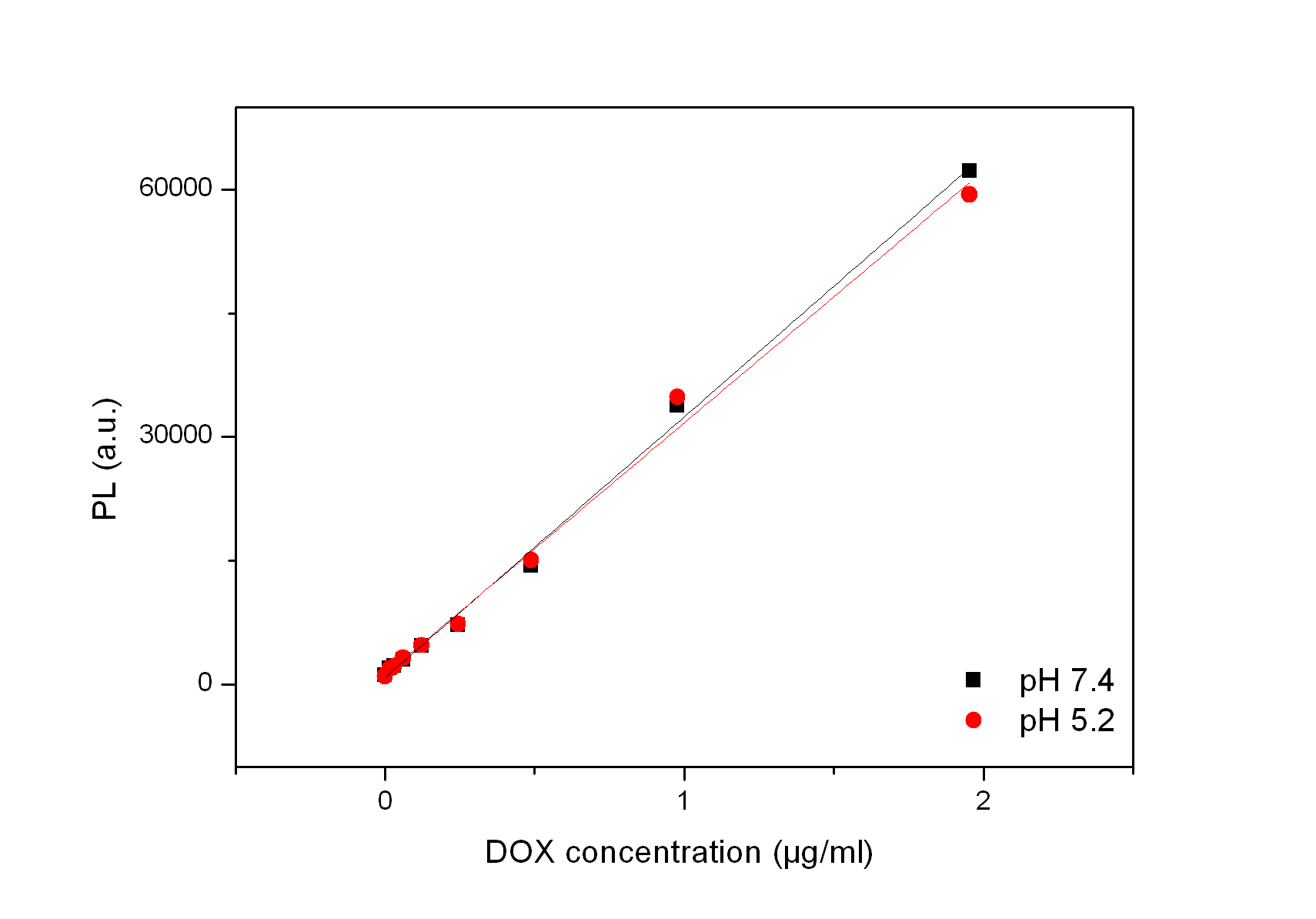
**

**Figure 2: shows the pore diameter distribution for different number of bilayers: A) non bilayers B) 2 bilayers C) 8 bilayers**

**
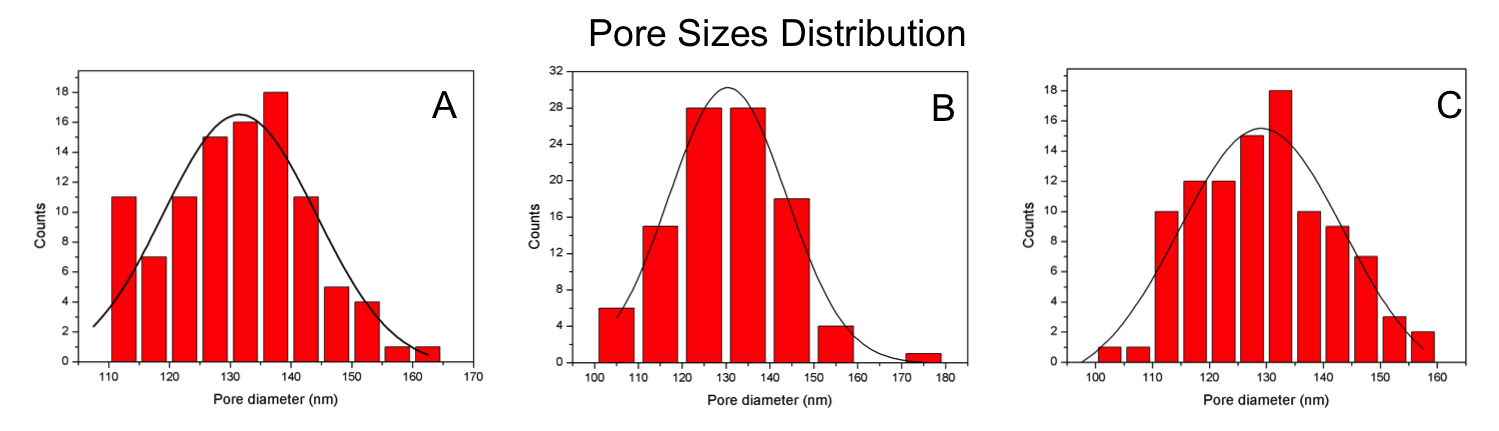
**

**Table 1 shows mean pore diameter for samples without polyelectrolyte bilayers, with 2 polyelectrolyte bilayers and with 8 polyelectrolyte bilayers.**

|  | Mean pore diameter (nm) | Standard deviation |
| --- | --- | --- |
| 0 bilayers | 131 | 11,30 |
| 2 bilayers | 130 | 12,70 |
| 8 bilayers | 130 | 11,93 |

**Table 2 shows different R-square for different fittings using different times. In this case the selected time was 30 minutes due to the highest R-squared values at this time.**

|  | | 30 minutes | 45 minutes | 60 minutes | 90 minutes | 120 minutes |
| --- | --- | --- | --- | --- | --- | --- |
| **First Burst Release pH5.2** | 8 bilayers | 0,99744 | 0,99351 | 0,99063 | 0,97674 | 0,97143 |
|  | 5 bilayers | 0,99716 | 0,99259 | 0,98948 | 0,97801 | 0,97505 |
|  | 2 bilayers | 0,99616 | 0,98601 | 0,97956 | 0,96166 | 0,95337 |
